# With some risk-taking and luck: A veterinarian’s adventures in viral immunology

**DOI:** 10.1371/journal.ppat.1007000

**Published:** 2018-07-05

**Authors:** Luis J. Sigal

**Affiliations:** Department of Microbiology and Immunology, Thomas Jefferson University, Philadelphia, Pennsylvania, United States of America; The Fox Chase Cancer Center, UNITED STATES

Like many other kids, I had already decided my future at the mature age of 5. Because I liked animals, I was going to be a veterinarian. Unlike most kids, I did not change my mind as I grew up, and 19 years later, I graduated with a veterinary degree from the University of Buenos Aires, Argentina. I opened a small practice in a semirural town near Buenos Aires. However, my practice was not as exciting as my childhood dreams. My days were filled with giving vaccine shots, rehydrating dogs suffering from parvovirus or distemper (most of which died), and performing the spine-chilling job of removing fly maggots from open wounds in the hot summer months. About 4 years into the profession, I decided that being a veterinarian bored me and that what really interested me was not to give vaccine shots but to find out how pathogens produced diseases and how vaccines prevented them. I learned that my veterinary school had just appointed a new professor of immunology, Marta Braun, who was starting a research program in viral immunology and vaccines against foot-and-mouth-disease virus in cattle. I knocked on Marta’s office door and told her that I was interested in doing research. Even though I had no research experience, I was lucky that she liked me and offered me a position as a teaching and research assistant. I took the risk, sold my practice, and from then on, my professional life centered on research in viral immunology. A couple of years later, Argentina reached the staggering annual inflation rate of approximately 3,000% (yes, 3,000!). Because the economic situation was so dire, my wife Norma and I decided to try finding a place abroad where I could do a PhD, hoping that, in the interim, the situation in Argentina would improve. Friends of Marta at the University of Nebraska recommended me to Dwane Wylie, an associate professor of immunology. For reasons I still do not know, Dwane decided to sponsor me to enter the PhD program and hired me as a research assistant without ever meeting me. I moved to Nebraska with Norma in July 1990 without having ever been there and started my PhD in September. Dwane gave me incredible freedom to pursue my interests. A great new discovery at the time was that CD8 T cells recognized viral antigen as peptides bound to a groove inside major histocompatibility complex (MHC) class I molecules. I found this fascinating and decided to focus my dissertation on understanding how influenza virus peptides interacted with MHC I, which was only obliquely related to Dwane’s own current interests. This brought me right into the field of antigen presentation.

As I was finishing my PhD in 1994, I wrote many letters (snail mail at the time) requesting postdoctoral positions to the big shots in antigen presentation. I was lucky that one of them, Kenneth Rock, then at the Dana Farber Cancer Institute and Harvard Medical School, answered and invited me for an interview. Ken liked me and offered me a position on the spot. A main interest in Ken’s lab was cross-presentation, a process whereby dendritic cells and macrophages take up exogenous antigens and present their peptides with their own MHC class I molecules. At the time, however, it was unknown whether cross-presentation was physiologically important or just an epiphenomenon. Indeed, we didn’t even know how CD8 T-cell responses to viruses were initiated. Using bone marrow chimeras and the poxvirus vaccinia virus (VACV, the smallpox vaccine), we showed that only cells of hematopoietic origin can initiate antiviral CD8 T-cell responses (we later extended this to other viruses), and by collaborating with Raul Andino, an Argentinean virologist specialized in poliovirus, we used mice transgenic for the human poliovirus receptor and Raul’s recombinant polioviruses to show that when hematopoietic cells cannot be infected, they use cross-presentation to take up viral antigens from other infected cells, thereby initiating the antiviral CD8 T-cell response.

My postdoctoral work with Ken made a splash and opened the door to establish my own lab at Fox Chase Cancer Center in Philadelphia as an assistant professor. I initially focused on solving some outstanding issues in cross-presentation using VACV, for which I rapidly obtained NIH funding. Yet the project was too much into cell biology, which was not what my research passions were. About 2 years after establishing my lab, at a small symposium in Madrid, Spain, I heard presentations by Antonio Alcami and Guna Karupiah on a poxvirus of the mouse known as Ectromelia virus (ECTV). This virus causes mousepox, a disease similar to human smallpox in some strains of mice but not others and, similar to smallpox, can be prevented by immunization with VACV. I immediately became hooked because ECTV offered a tractable model to study the genetic control of viral disease and to understand how a well-established vaccine protects a natural host. I made the risky move to start a new project in an area in which I had no experience, but I was lucky that the move paid off. Within a few years, I established a strong and well-funded research program using ECTV to understand fundamental problems in viral immunology. Using ECTV, my research team, now at Thomas Jefferson University in Philadelphia, has studied many different aspects of genetic, age-related, and vaccine-induced resistance to viral diseases, including the role of various mechanisms of host innate and adaptive immunity, as well as slick immune evasion countermeasures by the virus. So far, my scientific career has been an exhilarating adventure that I believe made important contributions to our understanding of the pathogenesis and immunology of viral infection, the very same interests that brought me into science when I was a young but bored veterinarian.

**Image 1:**
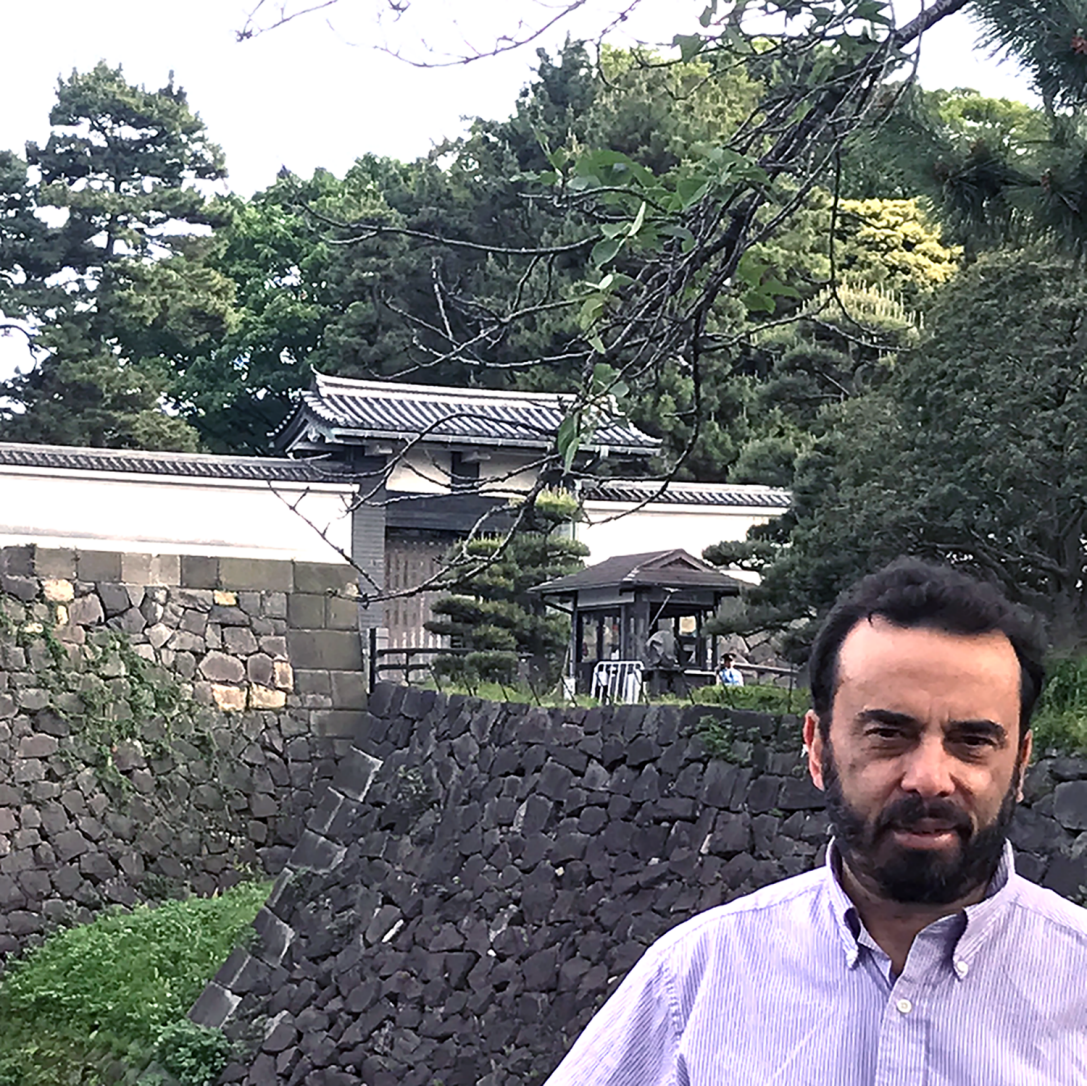
Luis J. Sigal.

